# Recommender systems use in weight management mHealth interventions: A scoping review

**DOI:** 10.1111/obr.13863

**Published:** 2024-11-13

**Authors:** Bianca A. de Castro, Sara M. Levens, Margaret Sullivan, George Shaw

**Affiliations:** ^1^ Department of Epidemiology and Community Health University of North Carolina at Charlotte Charlotte North Carolina USA; ^2^ Department of Psychological Science University of North Carolina at Charlotte Charlotte North Carolina USA; ^3^ The School of Information at Florida State University Tallahassee Florida USA; ^4^ Department of Health Management and Policy, School of Data Science University of North Carolina at Charlotte Charlotte North Carolina USA

**Keywords:** mobile app, recommender system, user engagement, weight management

## Abstract

The use of recommender systems in mobile health apps for weight control has grown, but user app uptake and engagement remain limited. The objective of our scoping review was to explore the influence of recommender systems on mHealth app user engagement, identify the theoretical frameworks that have been applied on digital health interventions designed for weight management, and examine the key aspects that support tailoring user engagement through recommender systems. Based on existing literature, we identified 13 articles on recommender systems for weight management. Themes emerged, including theoretical underpinnings, authors' domain knowledge, user motivation, and design. Most studies used constructs from the social cognitive theory. We found inconsistencies between authors' domain knowledge and the intervention's content, with few professionals from the health and psychology fields. Only 46% of articles measured user engagement, whereas gamification and tailored messages were common app features. Despite some positive weight change results, more attention is needed toward implementing behavior theory and other strategies to promote app user engagement. Future studies should more accurately measure user motivation and identify the best features and behavioral constructs to increase app user interaction. Larger studies with a more diverse population are needed to generalize findings and evaluate weight loss maintenance and physical activity habits among users of recommender system.

**Abbreviations**
mHealth apps: mobile health applications
RSs: recommender systems
PA: physical activity
SCT: social cognitive theory
SDT: self‐determination theory
TPB: theory of planned behavior
TTM: transtheoretical model

## 1 INTRODUCTION

The recommendation to eat healthy foods and exercise more often has become vital to reduce the obesity epidemic and the soaring financial healthcare expenditures in the United States. Although changes in human behavior are complex processes to implement,^1^ mobile health applications (mHealth apps) have been consistently employed and improved as valuable tools that can assist users in changing their habits, manage dietary intake, and increase physical activity (PA) levels.^2^, ^3^, ^4^, ^5^ Wellness apps are dominating the market, comprising approximately two‐thirds of all mHealth apps available.^6^ Despite their popularity and usability, poor user experience and motivation may be barriers that limit user engagement.^5^ Research has shown that addressing these barriers can improve user satisfaction and encourage more widespread and long‐term adoption of these tools.^7^ Therefore, heightened user adherence may facilitate crucial behavior changes toward healthier habits.

To engage users with mHealth apps, the systems should fulfill essential criteria for users, such as attractiveness and usability. App users seek software that is easy to use, has acceptable levels of data‐entry burden, and helps them achieve their goals.^8^ However, those features may be insufficient at ensuring continuing app engagement, user satisfaction, and/or adherence. A recent meta‐analysis has found that up to 80% of mHealth app users do not typically access the apps and sufficiently interact with them.^9^ Additionally, only 3.9% of participants use the apps for more than 15 days.^10^ The lack of engagement and utilization of these apps for extended periods might result from the absence of features that reduce information overload, as well as from missing adaptations that would meet users' needs and preferences, known as recommender systems (RSs).^11^

RSs are software tools based on machine learning techniques that offer personalized choices to users and suggestions supported by past individual preferences and behaviors.^12^ The use of RSs has been associated with better decision‐making and more user engagement,^11^ which can lead to success in change and behavior maintenance. Personalized digital advice can enhance and support healthier changes in individuals' diets^13^, ^14^ and PA,^15^, ^16^ which can better support medical suggestions and encourage healthier lifestyles. Techniques such as collaborative filtering, content, and knowledge‐based or hybrid recommenders^17^ make those systems collect and organize essential users' data to forecast suggestions they will more likely accept. For instance, some RSs can use past experiences of the individuals—like time, context of food consumption, or PA, to send reminder notifications, recommend similar healthier foods, or suggest exercises.^18^

To encourage behavior change, RSs in mHealth interventions must provide individualized and valuable recommendations in an accessible way and at a frequency that will engage users. This level of personalization will benefit if the mHealth intervention is grounded in behavior change theories that try to understand and explain the complexities of human behavior change.^19^ Although not always theory‐driven designed, some apps incorporate behavioral health constructs such as social support, self‐efficacy, and self‐regulation.^20^ The apps can use these constructs alone or, most commonly, in combination to support lifestyle changes and the development of healthy habits, including having a nutritious diet and appropriate weight. For example, despite the critical importance of self‐regulation in theory and practice, and the potential for mHealth apps to aid self‐regulation, long‐term self‐regulation remains a challenge for many mHealth app‐related interventions, highlighting the need to incorporate additional psychological constructs (such as self‐efficacy) and goal planning strategies that support longer‐term engagement.^21^

Engaging users in sustained app use demands mHealth apps with the functions capable of stimulating behavioral changes. According to social cognitive theory (SCT), self‐regulation is paramount to control human behavior, override previous habits, and move people over time toward the direction of their goals.^22^ However, self‐regulation cannot be achieved entirely without self‐monitoring, one of its subfunctions that allow people to monitor their behaviors (e.g., dietary intake) or health outcomes (e.g., body weight) and compare their progress with goals and previous experiences.^22^, ^23^ In other words, when individuals become aware of their eating or exercise patterns, this performance self‐monitoring assists them in understanding their reality and influences motivation to change habits. Additionally, some researchers have identified feedback and goal‐setting as crucial processes for helping individuals sustain motivation and adhere to behavioral changes.^24^, ^25^ These strategies provide individuals with regular evaluations of their progress, which is essential for supporting people in staying on track. Self‐efficacy is another critical psychological construct related to self‐regulation that is responsible for the feeling generated when people know they have the tools and the ability to complete a given task.^26^ These self‐efficacy beliefs affect people's choices, aspirations, efforts to achieve their goals, and motivation. To illustrate, previous research has associated self‐efficacy with healthier habits, for example, individuals with higher self‐efficacy have shown increased PA frequency compared with those with lower self‐efficacy.^27^ In addition to those constructs and techniques used in RSs in mHealth apps, there are recent trends in using game design elements, or gamification, to maintain engagement with health behavior apps.

Gamification employs different types of motivational strategies, using information presentation and rewards to harness the reward pathways in the brain and motivate particular behaviors.^28^ Individuals who are underactive or live with obesity have been shown to have over‐reactive reward networks that increase hedonic responses to food, making self‐regulation more challenging. Gamification, however, due to its affective and motivational potential, presents the possibility of harnessing an individual's overactive reward network to facilitate healthful behavior change.^29^ Thus far, gamification has been used predominantly on apps focused on diet and PA. Studies show that they effectively promote health behavior changes, considering that they share critical elements with other behavioral theories.^30^ Rewards through points or badges, setting challenges, narrative storylines, and avatar‐based self‐representation are some of the design elements used in apps with gamification.^31^, ^32^, ^33^ Studies have demonstrated that incorporating enjoyable elements into an individual's daily or less engaging activities—like regular physical exercise—can enhance their motivation and engagement levels. Subsequently, this can encourage healthier behaviors.

There are various theoretical frameworks and strategies used in mHealth with RSs, but the knowledge about which constructs and techniques are more effective in engaging users to facilitate healthful behavior change is limited and not precise. To our knowledge, no studies have comprehensively explored RSs' features and associated behavioral constructs embedded in mHealth apps for PA and diet control focused on weight management. To address this dearth in knowledge, we conducted a scoping review of experimental studies using weight management apps with RSs and characterized the apps through descriptive coding.^34^ We aimed to synthesize the recent knowledge about RS within mHealth apps developed for weight management to answer the following research questions: (1) What is known from the existing peer‐reviewed, published literature about RS' influence on users' weight management mHealth app engagement? (2) What theoretical frameworks have been utilized in mHealth apps with RSs developed for weight management?, and (3) What themes have been identified to support RSs in tailoring user engagement to mHealth apps for weight management?

## 2 METHODS

### 2.1 Selection criteria

Peer‐reviewed studies in the English language that included mHealth interventions with RS were included in this review. Participants were mHealth app users who aim to lose or manage weight through PA or dietary intake tracking systems, regardless of age, gender, or location. We considered articles with experimental and quasiexperimental study designs, including randomized and nonrandomized controlled trials, prospective and retrospective cohorts, and case–control studies. Descriptive and any type of reviews (systematic, narrative, scoping, meta‐analysis) were not included in our eligibility criteria.

### 2.2 Design and search strategy

We conducted a scoping review in conformity with the PRISMA‐ScR (Preferred Reporting Items for Systematic Reviews and Meta‐Analyses Extension for Scoping Reviews^35^) to detect interventions with mHealth apps developed with RS in which users were seeking weight management. Although generally recommended to enhance clarity and rigor, the study protocol was not registered because of initial time constraints. The literature search retrieved published studies from 2007 to 2022. The search strategy was conducted using Web of Science, PubMed, CINAHL, and ACM databases to identify articles on mHealth apps with RS used for weight management. A combination of the keywords (“recommender system” OR “recommender”; “weight management” OR “weight control” OR “weight loss”; “mHealth app” OR “mobile application”; “physical activity” OR “PA”; “intervention”) was adapted and used in each database.

After applying the search approach, the titles and abstracts of 3159 articles were screened by one author (BAC). Duplicate articles found in different databases and those that result in misclassification from keywords (e.g., “recommender” term not related to RS) were removed. Moreover, the bibliographies of 1512 articles were searched for additional sources, and seven new records were retrieved from the studies. To guarantee the compatibility with the defined criteria, a second thorough screening of the titles and abstracts was conducted, and three elements were observed: (1) mHealth apps described in study interventions for weight management; (2) RS associated with mHealth apps; and (3) mHealth apps with PA monitoring should not be exclusively related to performance or rehabilitation, but weight management or weight loss. The papers that did not fit the above criteria were removed, and 48 articles were eligible to be independently reviewed by two authors (BAC and GSJ).

**FIGURE 1:**
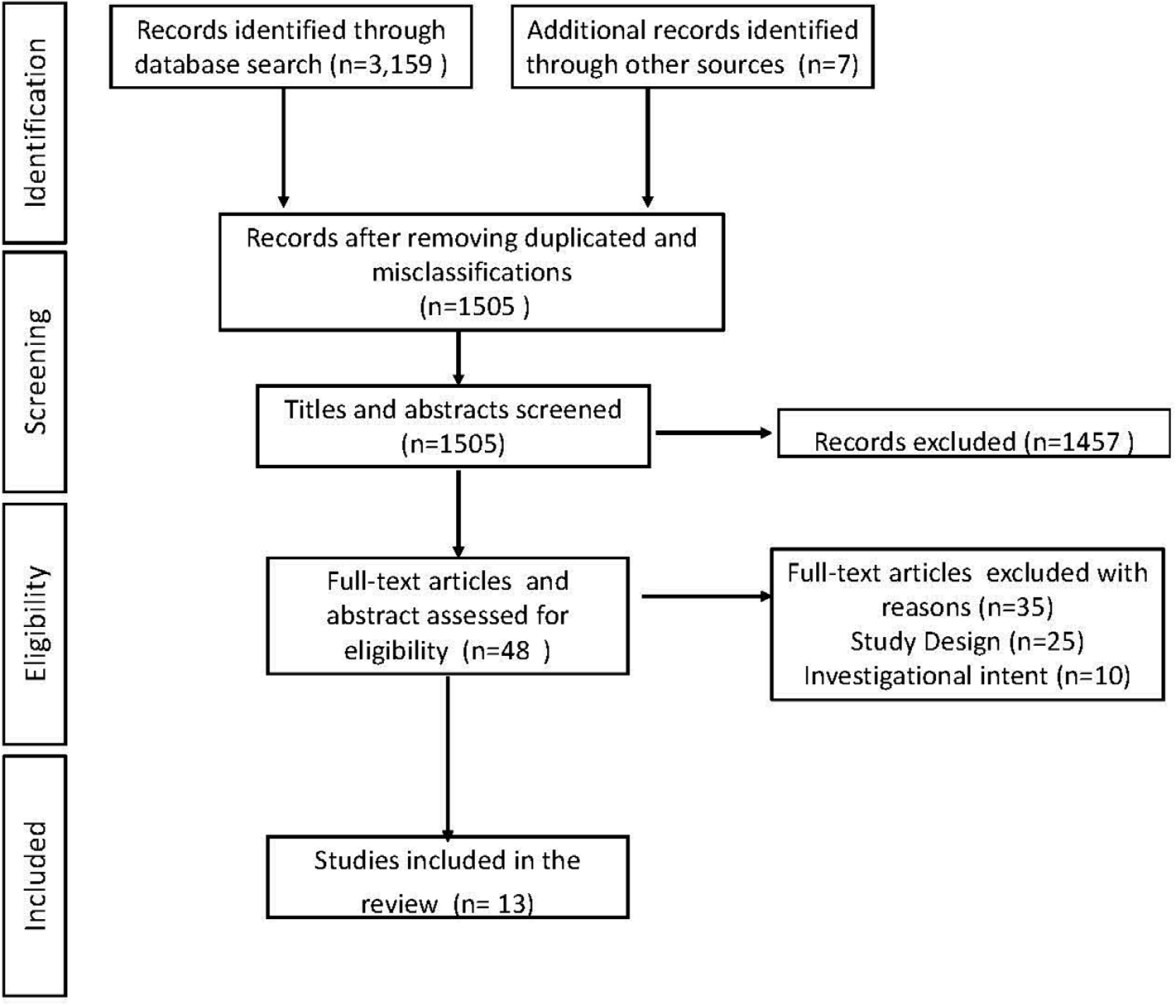
PRISMA‐ScR diagram.

During two meetings, the reviewers discussed the eligibility of the selected studies. Using R Studio software (version 1.4.1106), they performed interrater reliability testing with Cohen's kappa before thoroughly assessing the articles. Among the 48, the reliability between the reviewers was *k* = 0.96, and the percentage agreement was 98%, which supported a high level of mutual concordance. Next, a full‐text analysis of the final 48 studies jointly selected by the authors was performed. After removing 35 studies that, upon a thorough reading, did not meet the criteria for study design and the purpose of the research questions, a total of 13 articles remained for descriptive coding. Figure 1 illustrates the search process.

### 2.3 Data extraction

After the full‐text screening, the study details were extracted from the included articles utilizing the Excel program (version 16.71). One of the authors (BAC) conducted the initial data charting, while the other (GSJ) reviewed the document. The data collected included study details (authors, year, country, title, number of participants, design, and main outcomes) and mHealth app characteristics (RS details and concepts/theories that supported the technology).

## 3 RESULTS

The 13 included studies were published between 2010 and 2021, following an ascending tendency of increasing interest in the subject in the last 5 years (*n* = 8; 61%). Most studies were conducted in North America (*n* = 9; 69%), mainly in the US and European countries (*n* = 5; 38%), and among adults (*n* = 11; 84%). Most of the interventions were randomized clinical trials (*n* = 8; 61%), followed by cohort studies (*n* = 2; 15%), case–control studies (*n* = 1; 8%), experimental with a case study (*n* = 1; 8%), quasiexperimental (*n* = 1; 8%), and single‐case experiment design (*n* = 1; 8%). Table 1 briefly describes the studies' main characteristics.

**TABLE 1:** Characteristics of included studies.

Author/(year)	Country	Title	Study participants	Study design	Main outcome measures
Lee et al. (2010)	USA	Evaluation of a mobile phone‐based diet game for weight control	37 adults from an obese clinic assigned to a case/control group for 6 weeks	Case–control study	Fat mass, weight, BMI, and diet knowledge
Burke et al. (2012)	USA	Using mHealth Technology to Enhance Self‐Monitoring for Weight Loss: A Randomized Controlled Trial	210 adults (aged ≤ 59 years) with overweight/obesity randomly assigned to one of three groups	RCT	% weight change (baseline‐24 months) and adherence to diet self‐monitoring
Rabbi et al. (2015)	USA	Automated Personalized Feedback for Physical Activity and Dietary Behavior Change with Mobile Phones: A Randomized Controlled Trial on Adults	17 participants (aged 18–49 years) with low‐moderate PA levels randomly assigned into intervention/control group for 3 weeks	RCT	PA levels, caloric intake, adherence, and app recommendation acceptance
Rabbi et al. (2015)	USA	MyBehavior: automatic personalized health feedback from user behavior and preferences using smartphones	16 participants (aged ≥ 18 years) assigned to baseline/control conditions for 14 weeks	Single‐case experiment design (multiple baseline design)	Weight and calorie loss, users' intentions to follow suggestions
Hales et al. (2016)	USA	Social Networks for improving healthy weight loss behaviors for overweight and obese adults: A randomized clinical trial of the social pounds off digitally (Social POD) mobile app	51 adults with overweight/obesity randomized into control/intervention group	RCT	Weight loss and BMI
Zhou et al. (2018)	USA	Evaluating machine‐learning‐based automated personalized daily step goals delivered through a mobile phone app: Randomized controlled trial	64 adults' college employees assigned to intervention/control group	RCT	Change in daily steps and barriers to exercise
Hochsmann et al. (2019)	Switzerland	Effectiveness of a Behavior Change Technique‐Based Smartphone Game to Improve Intrinsic Motivation and Physical Activity Adherence in Patients With Type 2 Diabetes: Randomized Controlled Trial	36 sedentary adults (aged 45–70 years) with overweight/type II diabetes randomly assigned to intervention/control group	RCT	Intrinsic PA motivation, PA levels, and adherence
Zhao et al. (2020)	Canada	Effects of a Personalized Fitness Recommender System Using Gamification and Continuous Player Modelling: System Design and Long‐Term Validation Study	40 participants randomized into 4 groups: (1) gamified + personalized app, (2) gamified only, (3) personalized only, and (4) control group	Experimental study with a case study	PA levels, intrinsic motivation, and satisfaction
Gomez‐del‐Rio et al. (2020)	Spain	Health Promotion for Childhood Obesity: An Approach Based on Self‐Tracking of Data	45 children (aged 6–12 years) with obesity divided into control/intervention	A quasiexperimental, longitudinal prospective study	A new model and architecture for an activity recommender system
Afonso et al. (2020)	Portugal	A mobile‐based tailored recommendation system for parents of children with overweight or obesity: a new tool for healthcare centers	28 parents/caregivers of children between 3 and 6 years old with overweight/obesity randomized into intervention/control group for 4 weeks	RCT pilot study	Perceptions about child's weight status and knowledge about guidelines (fruits/vegetable/water consumption)
Sainz‐de‐Abajo et al. (2020)	Spain and Portugal	FoodScan: Food Monitoring App by Scanning the Groceries Receipts	109 rural residents (aged ≥ 70 years) with low technological knowledge	Prospective cohort	End‐user app evaluation and usability
Lugones‐Sanchez et al. (2020)	Spain	Effectiveness of an mHealth Intervention Combining a Smartphone App and Smart Band on Body Composition in an Overweight and Obese Population: Randomized Controlled Trial (EVIDENT 3 Study)	440 sedentary individuals (aged 20–65 years) with overweight/obesity	RCT	Weight loss, percentage of body fat, and BMI
Hu et al. (2021)	USA	Sustaining weight loss among adults with obesity using a digital meal planning and food purchasing platform for 12, 24, and 36 months: a longitudinal study	1740 adult app users with BMI > 30	Longitudinal retrospective study (no control group)	Nutriscore, 5% weight reduction

Abbreviations: BMI, body mass index; PA, physical activity; RCT, randomized controlled trial.

To capture data from the studies, we used descriptive coding. Through an inductive process and several revisions, the interventions were classified based on four themes: theoretical underpinnings, authors' domain knowledge area, user engagement/motivation, and RS design (Table 2).

**TABLE 2:** Selected articles classified into four themes.

Author/(year)	Theoretical underpinnings	Domain knowledge area	User engagement/motivation	RS design
Lee et al. (2010)	N/A	Health informatics, public health, and medicine	Daily app use: 8% participants; easy access/use and intention to use in the future: 58% users	Diet planner and game; avatar; manual or automatic diet/PA data entry
Burke et al. (2012)	SCT	Health systems and medicine	Adherence to self‐monitoring ≥ 60% intervention group; adherence to self‐monitoring predicted weight loss; 85% retention rate 24 months	Personalized daily message; goal setting; daily/weekly diet and exercise goals
Rabbi et al. (2015)	SCT, Learning Theory, and the Fogg Behavior Model	Information science, preventive medicine, and computer engineering	Intervention group: better adherence and positive rating vs. nonpersonalized suggestions by professionals; low‐effort suggestions increased self‐efficacy and motivation, reducing effortful behavior	Low‐effort automated recommendations from food log, PA records, environment, and past behavior; PA data entry automatic/manual (clustered by type/place); food data entry manual (pictures/label calories)
Rabbi et al. (2015)	SCT, Learning Theory, and the Fogg Behavior Model	Science and technology	Intervention group: higher adherence to PA and caloric intake suggestions vs. the control group with expert recommendations. Low effort and motivation are equally critical (Fogg Behavior Model). Increased motivation enhances low‐effort execution. Suggestions remain actionable in low‐motivation states.	Low‐effort automated recommendations from food log, PA records, environment, and past behavior; PA data entry automatic/manual (clustered by type/place); food data entry manual (pictures/label calories)
Hales et al. (2016)	SCT	Health promotion, computer science	Attrition rate = 12%. The social app: user self‐monitoring and engagement impacted motivation, leading to greater weight loss in the control group	App notifications (timed) and user‐to‐user messages
Zhou et al. (2018)	SCT	Industrial engineering and medicine	Attrition rate = 6%. Control group achieved higher goals with adaptative, personalized, and feasible PA recommendations, elevating self‐efficacy and motivation.	Push notifications: personalized step goals, performance comparisons
Hochsmann et al. (2019)	SDT	Health promotion, medicine, and technology	Game with personalized PA recommendations increased PA adherence over 24 weeks. Intervention group: increased intrinsic motivation	In‐game workouts with personalization (levels and rates of intensity), all adjusted based on user preferences and limitations
Zhao et al. (2020)	SDT, Hexad Model	Information technology, computer science	Personalized recommendations with player modeling and gamification increased user engagement and motivation.	Gamification and personalization using player modeling
Gomez‐del‐Rio et al. (2020)	Quantified self‐model	Educational sciences, computer engineering, and systems	RS enhanced user adherence by considering six aspects related to the user's point of view. Gamification enhances personalization, increasing user motivation. Long‐term improvement in healthy eating knowledge	Data collection from multiple sources. Recommendations: sports, active games, enhanced healthy habits, knowledge, and motivation tasks; gamification
Afonso et al. (2020)	TPB and SDT	Psychology and educational sciences, engineering	Attrition rate = 21%. Control group: parents showed higher engagement to the recommendations	Personalized video recommendations to parents about kids' weight status, food/beverage intake, PA, and sleep
Sainz‐de‐Abajo et al. (2020)	N/A	Engineering and computer sciences	66% reported app usability.	Tailored recommendations; data entry by scanning or adding grocery receipts
Lugones‐Sanchez et al.(2020)	N/A	Health and medicine	56% used the app ≥ 60 days; 60% considered the app easy to use	Personalized and integrated diet and PA recommendations and weight loss goals
Hu et al. (2021)	TTM	Health industry	Median enrollment = 25 months	Digital nutrition platform: food frequency questionnaire, healthy meal recommendations, personalized meal planning, and customized grocery discounts

Abbreviations: N/A, not applicable; RS, recommender system; SCT, social cognitive theory; SDT, self‐determination theory; TPB, theory of planned behavior; TTM, trans‐theoretical model.

### 3.1 Theoretical underpinnings

Thirty‐eight percent of the papers^36^, ^37^, ^38^, ^39^, ^40^, ^41^ explored interventions that used SCT with self‐monitoring, goal setting, self‐efficacy, or feedback constructs. In a previous study, researchers^36^ found that adherence to self‐monitoring was a crucial variation factor across the groups. Individuals in the intervention group who had positive and reinforcement messages toward self‐monitoring had a greater percentage of weight loss at 24 months (−2.32% [95% CI = −4.29, −0.35; *p* = 0.02]) compared with participants in other groups. Hales and colleagues^39^ combined self‐monitoring with daily notifications, messages, and rating system feedback from other users (social support), leading to positive behavior changes. Another study compared mHealth apps based on goal‐setting methods finding that adaptive goals outperformed fixed goals in increasing PA levels.^40^

Rabbi and colleagues^37^, ^38^ combined different behavioral theories to engage participants with the app. They used learning theory to assess users' skills to perform the action and the Fogg Behavior Model^41^ (FBM) to apply the SCT principle of self‐efficacy to the RS. Therefore, the app analyzed the proper context and time to create triggers that support low‐effort suggestions. These recommendations, when repeated by the users, reinforced their self‐efficacy and continually reduced the perception of effort needed to initiate an action, which positively impacted their motivation and results. Participants in the intervention group reported increased PA frequency and higher intentions to follow recommendations, even in low motivation states, compared with the control. However, the caloric intake difference between the groups was only significant in the 14‐week study.^38^ It is worth mentioning that although not a theory, the researchers used the trans‐theoretical model^42^ (TTM) to recruit participants. This model posits that people do not change their behavior decisively, but it takes a continuous process across six stages based on the person's level of motivation. Furthermore, only participants committed to change or actively taking the steps needed to modify their behaviors were selected for the interventions.

The TTM was also used in a longitudinal study to evaluate whether a digital nutrition platform that provides personalized recommendations for dietary intake and meal planning was helpful for sustained weight loss among users with obesity.^43^ The app aimed to embrace all participants' stages of behavior change, from precontemplation to maintenance. The nutrition quiz and meal planning were intended to target users in the initial stages in which they are aware of the problem but still unable to act. The app grocery component—with discounts and rewards—helped users to purchase healthier foods. Along with self‐monitoring, the app changed users' environment to facilitate a long‐term modification in their health behavior. The results showed that 22.4% of users had a sustained diet quality improvement and weight loss (≥5% initial weight) over a median of 25 months with similar results up to 36 months.

Two studies (15%) utilized self‐determination theory (SDT)^44^ for game‐based interventions promoting PA and personalized goals. Hochsmann et al.^45^ focused on sedentary type II diabetes participants and observed that the intervention group showed higher motivation and more in‐game walking (131.1 min/week; SD 48.7) and strength training (15.3 min/week; SD 24.6) compared with the control. Zhao and colleagues^46^ combined SDT and the Hexad model^47^ in their system and observed higher PA motivation, satisfaction, and engagement toward fitness activities in the intervention group compared with the control. Their system provided personalized recommendations based on users' preferences, personality type, and player‐type model. Game elements and motivational attributes were selected based on the player‐type model, and users were encouraged to invite friends and share achievements in social media profiles.

Afonso and colleagues^48^ used an RS supported by SDT and the theory of planned behavior (TPB)^49^ to provide recommendations to parents of kids with overweight or obesity. The intervention aimed to improve parents' perception of controlling their children's eating behaviors and “food parenting practices,” based on TPB predictors (beliefs, attitudes, and perceived behavior control) and SDT's self‐regulation. The intervention group showed increased guideline knowledge for water intake (*U* = 0.0, *p* < 0.001; large effect size) compared with the control, but no differences in perceptions of children's excess weight and fruit and vegetable guideline knowledge. On the other hand, Gomez‐del‐Rio and colleagues^50^ endorsed the quantified self (QS) field^51^ as a proposed model to address obesity in children. QS is a “school of thought,” collecting individual data from different aspects using technology.^52^ Despite being a model, not a theory, QS emphasizes self‐awareness through self‐monitoring and classifies the data using attitudes, behaviors, and emotions as parameters, suggesting that the model comprises aspects from the SCT. The app developed with a QS model showed slight improvements in children's health parameters and habits.

Two papers^53^, ^54^ (15%) lack clear information about the theoretical framework supporting the apps and interventions, but they seem to have a self‐monitoring approach. Lugones‐Sanchez et al.^55^ do not establish a specific theory foundation, but they mention key SCT constructs, including self‐efficacy and self‐regulation, through monitoring, goal setting, and tailored feedback. Compared with those who had diet counseling alone, females with BMI < 30 kg/m^2^ who accessed a mHealth app with a personalized diet and PA suggestions and a smart band had greater weight loss (−1.97 kg, 95% CI −2.39 to −1.54) and changes in body fat composition (−1.84 kg, 95% CI −2.48 to −1.20).

In summary, a considerable proportion (69%) of these studies have mentioned the theoretical framework that guided the development of their RS interventions, and the most common elements were self‐monitoring (77%), goal setting (54%), self‐efficacy (46%), and positive reinforcement/feedback (23%).

### 3.2 Domain knowledge area

The researchers' domain knowledge was mixed, with collaboration of professionals from the medicine, computer science, and engineering fields. Approximately 38% of the interventions were conducted by researchers from health/medicine and computer science areas^36^, ^37^, ^39^, ^44^, ^53^; 15% from health/medicine^43^, ^55^; 15% from science and technology^38^, ^46^; 15% from engineering and educational sciences^48^, ^50^; 8% from engineering and computer sciences^54^; and finally, 8% from engineering and medicine.^40^ Interestingly, only one study^48^ (8%) had a researcher from the psychology field.

### 3.3 User engagement/motivation

Among the articles, 38% defined measures to evaluate user engagement and motivation.^36^, ^37^, ^38^, ^43^, ^46^ For instance, Burke et al.^36^ reported that more app users achieved ≥60% of adherence to self‐monitoring than those in the control group, whereas Horchsmann et al.^44^ implemented an intervention to support intrinsic motivation elements (e.g., interest/enjoyment and perceived competence) and found a significant motivation increase in the intervention group (IMI total score (+6.4 [SD 4.2; *p* < 0.001] points) compared with a decrease in the motivation in the control (−1.9 [SD16.5; *p* = 0.623] points). Moreover, Zhao et al.^46^ found that using personalized recommendations and gamification increased users' motivation (F3,36 = 22.49; *p* < 0.001), satisfaction (F3,36 = 22.12; *p* < 0.01), and preference (F3,36 = 15.0; *p* < 0.001) compared with using one strategy alone.

Although some results were not established as outcomes beforehand, 46% of the studies reported elements related to user engagement and motivation, which contributed to both user adherence to the app and recommendations, thus facilitating users' weight management. For example, in the studies conducted by Sainz‐de‐Abajo et al.^54^ and Lugones‐Sanchez et al.,^55^ 66% and 60% of the participants, respectively, reported that the app with an RS was accessible and easy to use. In comparison, Lee et al.^53^ found that 58% of the participants reported accessibility of the app, interesting content, and an intention to use the app in the future. Furthermore, Hales et al.^39^ described that the user‐to‐user messages, as a component of social support, were essential in enhancing user self‐monitoring and engagement, encouraging diet and PA change behavior. Alongside the constructs of self‐monitoring and social support, Rabbi et al.^38^ emphasized self‐efficacy, which was increased by the low‐effort personalized app suggestions that the participants in the control group received. As a result, the participants showed a higher engagement with the recommendations and evaluated them more positively.

### 3.4 Recommender system design

Gamification features were found in 31% of the articles.^44^, ^46^, ^50^, ^53^ Different game design elements were used to foster human motivation and performance regarding weight management. Lee et al.^53^ used a three‐dimensional image representing the user's body shape that could help them to simulate their current and future body status, thus tracking their achievement. In contrast, Hochsman et al.^44^ incorporated their game rewards and feedback on performance to continue motivating users. The in‐game workouts were suggested based on individual preferences and limitations to recommend feasible and realistic exercise goals because overwhelming targets would negatively impact motivation and PA consistency. In another study, Zhao et al.^46^ implemented in‐game personalized challenges based on the classification of player type, as well as a customized storyline, and the addition of recommendations to exercise in several user contexts. These features together helped long‐term (60 days) user engagement and motivation for practicing fitness activities. Customization of recommendations using grocery information was implemented in two additional studies,^43^, ^54^ which accounted for 15% of the papers. Sainz‐de‐Abajo and colleagues^54^ conducted an app intervention where users could add or scan their grocery receipts. As a result, 49% indicated that personalized healthy meals based on the ingredients/foods they purchased helped with diet control. Conversely, Hu et al.^43^ implemented a study where the app gathers user food preferences and behaviors to provide healthy meal recipes and grocery discounts for healthy and cheap products. The RS helped users to plan their online food purchases with better choices while saving money.

Common system features encompassed tailored daily messages or notifications to support diet self‐monitoring,^36^, ^37^, ^38^, ^39^, ^40^, ^55^ progressive PA/diet goals,^36^, ^37^, ^38^, ^40^, ^55^ user‐to‐user messages to enhance social support,^39^ and manual food entry with the support of large food databases.^36^, ^39^, ^53^, ^55^ In addition, one mHealth app in both selected interventions^37^, ^38^ allowed photos and food labels to assist with food logging. These designs showed an improvement in user weight management and motivation compared to users not exposed to apps with RSs. In contrast to those features, one intervention (8%) presented personalized recommendations using video format instead of messages,^48^ and the parents from the control group showed higher motivation to help their kids with behavior change compared with those in the comparison.

Overall, the themes and results listed above demonstrated the benefits and emerging utility of RS for individuals with overweight or obesity who want to manage their weight. Most of the observed changes in the outcome measures, such as weight loss, BMI reduction, PA levels, and intrinsic motivation, indicated that using RS could be an efficient tool to improve users' lifestyles and help engagement and adherence to digital weight control interventions.

## 4 DISCUSSION

This study set out with the aim of assessing the current knowledge in the published literature regarding RSs in mHealth apps for weight management, specifically focusing on their influence on user engagement, the theories embedded in these apps, and the common themes that contribute to improving user engagement through RSs. The results of this review indicate that RSs were primarily employed to boost user engagement in the apps, with the goal of achieving more effective intervention results, whether or not these results were compared with those provided by standard recommendations. Another key finding is that theories or theoretical constructs to support RSs and their functions are frequently present in the mhealth apps reviewed. However, perhaps the most interesting finding is that the descriptive coding revealed four interconnected common themes (i.e., theoretical underpinnings, user engagement/motivation, domain knowledge, and design). Despite the implementation of psychological theories and strategies to enhance engagement, a noticeable inconsistency in researchers' domain knowledge was identified, which could potentially undermine the effectiveness of the interventions. This inconsistency may be attributable to the absence of psychologists in the RS development and intervention.

To gain a deeper understanding of the dynamics surrounding these digital interventions, the thematic analysis conducted offers a comprehensive framework to evaluate the use and effectiveness of RSs in tailoring user mHealth app engagement for weight management. The following discussion delves into the specific themes identified, starting with the theoretical underpinnings that supported the implementation of RSs.

### 4.1 Theoretical underpinnings

Although the vast majority of the studies conveyed information on theoretical frameworks, psychological theories that seek to explain and influence human behavior were the most used in the context of RSs. These findings align with prior studies highlighting the importance of behavior change theories in supporting weight management interventions. SCT, for example, has been extensively used in health promotion, given the consideration of individual behaviors and the environment in which individuals perform their behaviors.^19^ As an essential component, self‐monitoring increases people's awareness of their target behaviors and the factors that facilitate or prevent them from achieving these goals. When self‐monitoring is supported by a combination of self‐regulation techniques, such as goal setting and feedback, weight control interventions seem more effective than those that use one technique alone.^20^, ^56^, ^57^ Although some studies have failed to describe theoretical frameworks, this current review found that the majority of the mHealth apps with RSs for weight management utilized SCT or some of its constructs (i.e., self‐monitoring, goal setting, self‐efficacy, and reinforcement/feedback) to address diet and PA among the participants. These results support the work of previous reviews that found a positive correlation between self‐monitoring of weight, dietary intake, and PA frequency^20^, ^36^, ^57^, ^58^, ^59^; self‐monitoring and goal setting,^60^ self‐monitoring and self‐efficacy^37^; and self‐monitoring and feedback^23^ with weight loss interventions.

Surprisingly, none of the studies addressed regular self‐weighing, and there was no clear guidance on the optimal frequency and duration for self‐monitoring. These findings resonate with mixed results reported in other studies.^36^, ^58^ Moreover, little attention has been given to the environment, despite being one of the main focuses of the SCT and an important area of recent research. Only four studies have addressed the environment using contextualized suggestions,^37^, ^38^ an online food purchasing environment,^43^ and social support.^39^ This fact might explain the modest results in weight loss in most of the selected studies.

Although interventions among adults were predominant in the selected studies, it is important to recognize that weight management strategies, whether traditional or digital, must be tailored to participants' age or life period due to their unique metabolic, hormonal, and cognitive differences. For this reason, both dietary and PA recommendations, as well as the underlying theoretical constructs within the RSs, should be adapted to account for these individual variations in order to achieve the best possible outcomes. Despite reporting positive results from their program with an RS based on the QS user model and elements from SCT, evaluating RSs created for children is a complex task.^50^ These algorithm‐driven programs have been overlooked in the child‐centric literature, although children use RSs in a variety of situations.^50^ Additionally, programs widely use SCT constructs to improve nutrition and PA levels among children to prevent obesity. However, weak evidence has been reported linking effective weight management in kids and the use of behavior change theories,^61^ due in part perhaps to the significant role that parental eating habits play in child eating and weight management. Therefore, more research is needed to explore whether RSs based on SCT have the same potential for impacting child and adult behavior change. The results reported by Gomez‐del‐Rio and colleagues^50^ should be carefully considered because they may reflect a combination of strategies utilized rather than solely attributed to the app with an RS.

Lastly, it is worth mentioning that a more recent model of behavior change (COM‐B model) has been used to understand people's health behavior and measure the effectiveness of interventions to increase kids' PA as well as eating behaviors among young adults using digital interventions.^62^, ^63^ The model considers existing behavioral theories and hypothesizes that capability, opportunity, and motivation are the interactive components to influence someone's behavior.^62^, ^63^ Despite its crescent application, none of the articles have mentioned the model, demonstrating a lack of behavior change domain expertise in the design of weight management interventions featuring RSs in mHealth apps.

### 4.2 Domain knowledge

Although the use of behavior change theories is important to understand and drive strategies aimed at effectively changing people's behaviors, only one study included a researcher in the field of psychology. Although the application of psychological theory or models outside the discipline speaks positively to the applicability and robustness of these theories/models in the context of weight management and health behavior change, there is inherently higher risk and missed opportunity in nonpsychologists leading research based on psychological theories without the representation of psychological scientists on the research team. The absence of a psychology expert to assure that theories are interpreted and implemented correctly increases the likelihood of errors in these areas and may also suggest a lack of attention in implementing the interventions and interpreting the results with regard to key psychological constructs. The field of psychology includes domain expertise not only on key theories of self‐monitoring and behavior change but also in the behavior of the user, including their motivations, cognitions, habits, personalities, and emotions. In addition, the principles of gamification, a popular and effective RS design, are also based in psychology, highlighting another area where psychological domain expertise could improve RS mHealth applications. A lack of psychological expertise can therefore increase the potential of missing, misunderstanding, or misattributing nuanced findings, which in the context of RS mHealth can lead to wrong conclusions about the influence of a psychological construct with potential real‐world consequences.

Similarly, despite the urgency to control weight to address the obesity epidemic and all disease‐associated problems, less than half of the mHealth interventions with recommendations for diet and PA involved researchers from medicine or health. It is possible, however, that some of the academics listed as authors might have a health‐related degree or previous experience. This domain knowledge gap contrasts with an earlier systematic review of mHealth apps in weight management.^23^ The researchers found that among all the 22 randomized‐controlled trial studies selected, at least one of the authors presented health‐related knowledge. To design and implement effective mHealth interventions, it is essential to have representation from the key areas of domain expertise.

In order to successfully integrate and develop RSs into effective weight management apps, it is paramount to combine expertise from various fields. For instance, researchers from technology and computer sciences can contribute with data processing techniques and algorithms, behavioral experts and psychologists provide insights about user motivation and behavior change, whereas researchers from health and medicine ensure that the app recommendations are relevant and aligned with the most updated health guidelines. The observed inconsistency in researchers' domain knowledge across the studies also highlights a significant missed opportunity in mHealth RS research, as mHealth RS applications are an ideal space for interdisciplinary team science to thrive. If the authorship teams of the studies included in this review also reflect the disciplinary make‐up of the grant team that received funding to conduct the research, then the apparent domain knowledge inconsistency found across the intervention studies with mHealth RSs is even more concerning. Future mHealth RS research would greatly benefit from building more interdisciplinary research teams with domain knowledge that includes expertise in health and wellness, nutrition, and psychology. The importance of carrying out research with scholars from different fields and points of view was evident during the COVID‐19 pandemic, where several studies and advances were obtained with the cooperation of a considerable number of scientists from different areas and across countries.^64^, ^65^, ^66^ Additionally, online research partnership makes it easier to connect and interact with scientists regardless of their location, and the integrated knowledge across fields can spark significant advances in research. One common way to encourage this collaboration is through universities' policies and structural changes.^67^ Universities can offer graduate students' workshops and courses on the development of collaborative research and encourage integrated research through funding and events. A study among 156 American universities^65^ found that the institutions more structurally committed to interdisciplinary research achieved a higher number of research publications and received more grants from the National Institutes of Health (NIH). Therefore, given the relevance of weight control and the evolving mHealth app development, the complexities involved in creating development of efficient, effective, and engaging RSs to promote desired health outcomes amid the rising global obesity epidemic make collaborative teams crucial to expanding solutions to this public health threat.

### 4.3 User engagement and motivation

Motivation and engagement of app users showed mixed and inconsistent results. Only two studies^44^, ^46^ measured users' internal motivation levels using a short version of the Intrinsic Motivation Inventory (IMI), a standard instrument to assess someone's motivation, including motivation to exercise. The implementation of some features, such as gamification,^44^, ^46^ avatar,^53^ and easier forms of inserting dietary intake, such as incorporating scanning food labels or taking pictures,^37^, ^38^ has the potential to impact interaction and motivation, but none of these articles have properly justified those. Additional studies mentioned usability,^54^ adherence to self‐monitoring,^36^ acceptability,^37^, ^38^ and satisfaction^46^ as primary or secondary outcome measures, but no intervention used standard tests to evaluate them. Despite those outcomes being related to user experience and, by extension, user engagement, they might not fully capture user motivation in a broader sense. In addition, a desirable behavioral outcome would be daily use of the system, but few interventions reported the user's frequency of access. In Lee's study,^53^ for instance, most participants (75%) only accessed the system once a week, which shows a problem with user engagement and might have impacted the interpretation of the findings. Those results confirm the need to implement more solid theoretical bases when developing these RS apps and deciding to evaluate them in empirical studies that seek to change human behavior. Clearly, we can infer that the implementation of RSs in mhealth apps has the potential to enhance engagement, thereby impacting user motivation to some extent. However, without a more comprehensive evaluation—using quantitative tools such as the IMI, or combining it with qualitative measures, through feedback interviews or surveys to capture information, such as enjoyment, ease of use, and perceived value—it remains unclear whether the presence of RSs in weight management apps is sufficient to stimulate and sustain motivation to effectively galvanize health behavior changes.

### 4.4 Design

Various features were developed to enhance personalization in the studies. For example, game design elements have become popular in nongame contexts such as wellness and health environment, incorporating principles from health behavior theories. However, it remains unclear how those elements impacted behavior changes. Although a recent review^68^ has shown that using an avatar effectively elicited healthy behaviors, only one intervention^53^ used the component without attributing any effect to it. This finding suggests that the avatar representing the user's body image might not be relevant to behavior change or representative of individual self‐perception image. The storytelling and the inclusion of challenges into the game seemed to have played an important role in users' adherence to PA and engagement with the system^44^, ^46^ as it may have made the PA more playful and pleasurable. These results aligned with those from Johnson and colleagues^69^ whose systematic review of gamification for health found empirical evidence on the effectiveness of applying gamification to promote health behaviors. Nonetheless, because the interventions selected in this current study also contain behavior theory elements, we cannot identify to what extent the gamification features contribute to their results.

Another critical design was the use of reinforcement messages from the system itself or among users fostering self‐monitoring compliance and facilitating social support. As mentioned, people need tailored feedback to identify their current status and the actions required to pursue their goals. Reminding users that they have not logged their meals or exercise or even praising them for doing so is an important method to engage people in self‐monitoring. Moreover, the rating system messages sent from other users seemed to provide emotional support among participants and played a role in better outcomes. This finding is consistent with reported positive associations between social support and user engagement toward diet and exercise.^70^, ^71^, ^72^ Yet, the selected articles and literature do not define the ideal number of messages to be sent in order to achieve the best level of user engagement.

Unlike most studies in which users logged their daily meals, two studies^43^, ^54^ presented systems that offered meal planning and recipes by scanning labels of purchased foods or creating discount coupons for online grocery purchases. These features helped increase users' nutrition knowledge and provided more tools for people to control their diets, considering their economic power and preferences. This is important because several mHealth apps focus on people's behavior and ignore their food literacy, social and economic status, and other factors that may contribute to unhealthy options. Although it is well‐known that diet and PA are the main effective recommendations for maintaining a healthy weight, the interventions using mHealth apps that personalize both PA and diet did not show consistent and significant weight loss results. Except for Burke's study,^36^ where the participants lost 5% or more of their baseline weight, all other interventions resulted in a small percentage of weight or total weight loss.

Several possible reasons may explain those results, such as the app did not provide challenging or appealing goals, food or PA entry data burden, and external life events. The findings support the premise that RS helps promote behavior change and healthier habits for controlling weight. However, these results need to be interpreted with caution, given that we are not assessing the quality of the studies, the interventions had different methodologies, and the majority of them utilized small samples during a short‐term period. They also included a prevalence of highly educated, White females. Additionally, the studies did not account for income or other social determinants of health that may prevent people from following a healthy diet or practicing PA.

### 4.5 Limitations

This review has a few limitations. First, during the search process, the query terms were tailored according to the database to find articles that fit our eligibility criteria. This approach possibly created gaps in the search and loss of information. Second, the lack of an additional reviewer during the entire analytical process may have led to selection bias when the articles were chosen. Third, because we selected only experimental studies, we missed some details about the apps and RSs specifically described by other articles that were not assessed, including the RS type and the theoretical development specificities. Even though a few studies did not report one or more topics in our result section, we cannot assure that the themes were not mentioned in other related research articles. Moreover, one study^50^ combined a mHealth app with several strategies for promoting health behavior change among children with obesity. However, the absence of a control group and the variety of approaches and technological resources utilized prevent us from attributing the results of the intervention exclusively to the RS. Another limitation concerns the domain knowledge of the authors. Because we only evaluated the credentials provided in the selected studies, we may have missed the authors' previous experiences and background, which could lead us to incomplete conclusions about their expertise in psychology or health. Finally, gray literature and review articles were not consulted, possibly omitting relevant information.

## 5 CONCLUSION

The purpose of the current study was to identify and examine the body of literature regarding mHealth apps with RSs for promoting weight control management through changes in dietary behaviors and PA. Although not a comprehensive review, our findings provide insight to assist further studies in the mHealth and wellness area. Despite some positive results, more attention is needed toward implementing behavior theory components and other strategies to promote user engagement in RS apps to manage weight. Developers should better identify the most effective and evidence‐based designs to increase health app adherence. The interventions should include health and psychology expert members, as well as users' motivation measures and identification of the reasons that prevent people from complying with the personalized suggestions. Additionally, the studies should account for other social determinants of health that might impact people's food choices and PA levels. Further studies on the topic are warranted that include extended intervention periods and with representative, generalizable sample sizes.

## CONFLICT OF INTEREST STATEMENT

The project team members declare that they have no conflicts of interest related to the subject matter to report.
